# An unusual case of congestive heart failure in the Netherlands

**DOI:** 10.1099/jmmcr.0.005142

**Published:** 2018-03-06

**Authors:** Marjolein C. Persoon, Olivier C. Manintveld, Femke P. N. Mollema, Jaap J. van Hellemond

**Affiliations:** ^1^​ Department of Medical Microbiology and Infectious Diseases, Erasmus University Medical Center, Rotterdam, The Netherlands; ^2^​ Department of Cardiology, Erasmus University Medical Center, Rotterdam, The Netherlands; ^†^​ Present address: Department of Internal Medicine, Haaglanden Medisch Centrum, The Hague, The Netherlands.

**Keywords:** heart failure, *Trypanosoma cruzi*, Chagas disease

## Abstract

**Introduction:**

Chagas disease is caused by infection with the protozoan *Trypanosoma cruzi.* It is endemic to the American continent due to the distribution of its insect vectors. The disease is occasionally imported to other continents by travel of infected individuals. It is rarely diagnosed in the Netherlands and exact numbers of infected individuals are unknown. Clinical manifestations can start with an acute phase of 4–8 weeks with non-specific, mild symptoms and febrile illness. In the chronic phase, it can lead to fatal cardiac and gastro-intestinal complications.

**Case presentation:**

We describe a case of a 40-year-old man with end-stage cardiomyopathy due to Chagas disease. He lived in Surinam for more than 20 years and had an unremarkable medical history until he was hospitalized due to pneumonia and congestive heart failure. Despite antibiotic treatment and optimizing cardiac medication, his disease progressed to end-stage heart failure for which cardiac transplantation was the only remaining treatment. A left ventricular assist device (LVAD) was implanted as a bridge to transplantation. Tissue analysis after LVAD surgery revealed ongoing myocarditis caused by Chagas disease. Based on a literature review, a scheme for follow up and treatment after transplantation was postulated.

**Conclusion:**

Chagas disease should be taken into account in patients from endemic countries who have corresponding clinical signs. Heart transplantation in patients with Chagas cardiomyopathy is accompanied by specific challenges due to the required immunosuppressive therapy and the thereby increased risk of reactivation of a latent *T. cruzi* infection.

## Introduction

**Fig. 1.**
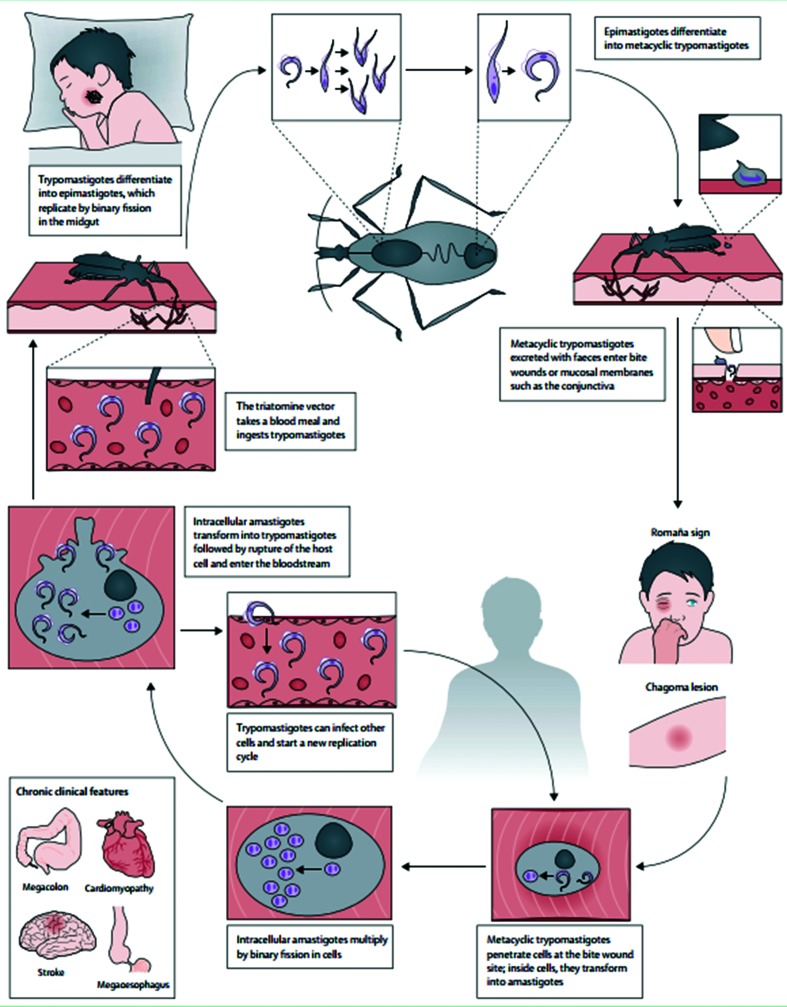
Life cycle of *Trypanosoma cruzi* (figure courtesy of Dr Perez-Molina [4]).

Chagas disease is a neglected tropical disease caused by the protozoan *Trypanosoma cruzi.* It is a major public health problem in Latin America, in particular affecting individuals in low socioeconomic circumstances [1]. A blood-sucking insect of the subfamily Triatominae is the primary vector in parasite transmission to humans. These insects are also called ‘kissing bugs’ due to bites that occur on the face as a result of the insects' affinity with exhaled carbon dioxide. Other routes of transmission include mother-to-child transmission, mainly caused by maternal parasitaemia, transmission through transfusion of blood products and solid organ transplantation. Less common routes of transmission are oral transmission through contaminated food and drink and laboratory accidents [2–4]. The life cycle of *T. cruzi* is shown in Fig. 1. Due to the geographical distribution of the insect vectors, the disease is only endemic to the American continent, where it is estimated that approximately 8 million people are infected. However, with increased human migration, individual cases are imported to other continents. It is estimated that over 10 000 people die annually due to Chagas disease [4].

After infection, most patients remain asymptomatic. However, non-specific, mild symptoms and febrile illness can occur in patients during the acute phase of the disease, 4–8 weeks after infection. At the inoculation site an inflammatory response (inoculation chancre) may become visible after an incubation period of 5–7 days. Inoculation through conjunctiva after scratching the site of the bite can lead to unilateral periorbital oedema, which is referred to as Romaña sign. The acute phase generally resolves spontaneously and severe acute disease, such as acute myocarditis and meningoencephalitis, is rare.

Without treatment, the acute phase is followed by a chronic phase, which is lifelong [5, 6]. The majority of patients remain asymptomatic and have no abnormalities on chest radiography or electrocardiogram. This stage is defined as the indeterminate phase of Chagas disease. It is estimated that 10–30 years after infection 30–40 % of infected patients will develop clinical manifestations, mainly cardiomyopathy and gastro-intestinal complications. Symptoms of the gastrointestinal tract can include dysphagia, odynophagia, oesophageal reflux, weight loss, aspiration, cough and regurgitation. Development of a megacolon is characterized by severe constipation and may result in faecalomas which can cause intestinal obstruction, volvulus and bowel ischaemia. Cardiac abnormalities mostly involve the conduction system and myocardium. Early signs may include left anterior fascicular block, right bundle branch block and segmental left ventricular wall motion abnormalities. Later signs are bradycardia due to sinus node dysfunction, atrioventricular blocks, ventricular tachycardia, complex ventricular extra-systoles, progressive dilated cardiomyopathy with congestive heart failure, apical aneurysms and emboli as a result of an aneurysm or dilated ventricle [7, 8].

Nifurtimox and benznidazole are the main drugs used for anti-trypanosomal treatment, but both drugs have substantial side effects. In general, benznidazole is better tolerated and has fewer drug–drug interactions. Hence, nifurtimox is mainly used in patients with insufficient response to benznidazole. Neither drug is registered in the Netherlands and the drug of choice has to be imported from abroad [9–11]. Treatment with trypanocidal drugs is always recommended in acute or congenital infection with *T. cruzi*, and in chronically infected children (<18 years old). Current consensus is to offer trypanocidal treatment also to adults in the indeterminate phase of Chagas disease and to those with mild-to-moderate disease. For patients older than 50 years treatment depends on severity of the infection and presence of other diseases.

In the Netherlands Chagas disease is non-endemic and rarely diagnosed. Exact numbers of infected individuals are not available but a study in 2011 estimated the number of individuals in the Netherlands with specific antibodies against *T. cruzi* to be between 726 and 2929 [6]. The vast majority of these people originate from Surinam, which is a former Dutch colony. However, a study performed in 2013 by Slot *et al*. detected no unrecognized patients by screening for IgG antibodies to *T. cruzi* among asymptomatic blood donors in the Netherlands with the following risk factors for Chagas disease: birth in an endemic country for Chagas disease, having a mother born in an endemic country or having lived in an endemic country for more than 6 months [12].

We present a case of cardiomyopathy due to Chagas disease in the Netherlands and describe the clinical symptoms, diagnostics and treatment options.

## Case report

In July 2014 a 40-year-old male was referred to the Erasmus University Medical Centre in Rotterdam, the Netherlands, for a second opinion. His complaints included severe dyspnoea and signs of congestive heart failure despite heart failure treatment.

His medical history included a pneumonia a month earlier for which he was admitted to a local hospital and treated with antibiotics. Subsequently he was seen by the cardiologist due to signs of congestive heart failure, ventricular tachycardia, right bundle branch block and a blood clot in the apex of the left ventricle for which cardiac medication and anticoagulants were started. The preliminary diagnosis of inflammatory cardiomyopathy due to infection was made.

The patient was born in Surinam and he had no medical history of note until the pneumonia in 2014. He had lived in the Netherlands for about 20 years. He did not drink alcohol, used no drugs and stopped smoking in June 2014 after smoking a few cigarettes a day for several years.

At admission in our hospital he presented with complaints of fatigue, swollen ankles and progressive dyspnoea for 6 months. The dyspnoea exacerbated with effort and limited his action range to only 10 m of walking. He slept in an upright position due to dyspnoea when lying flat. On physical examination his temperature was 38.5 °C, his blood pressure was 95/70 mmHg, central venous pressure was elevated and there was some oedema in the ankles. In addition to cardiac medication, a left ventricular assist device (LVAD) was implanted in September 2016 to provide mechanical circulatory support until transplantation.

## Investigations

A chest radiography showed an enlarged heart and a possible pneumonia of the right lower lobe. Echocardiogram showed impaired left ventricle function, dilatation of the left atrium, severe mitral valve insufficiency and moderate tricuspid insufficiency. In the apex of the left ventricle a blood clot was detected. Magnetic resonance imaging of the heart showed a left ventricle which was severely dilated and impaired left and right ventricle function.

Histological examination of a myocardial biopsy, taken after LVAD implantation, showed lymphocytic inflammatory infiltrate, indicating ongoing myocarditis. Serological and PCR tests for viral and bacterial causes of a myocarditis were negative, as well as bacterial blood cultures, syphilis and *Toxoplasma* serology. However, serological tests for the detection of antibodies against *T. cruzi,* performed by the Academic Medical Centre in Amsterdam (AMC), were positive. The positive results of a dipstick and immunofluorescence antibody test (1 : 320 with cut off reference value of 1:≥80) were subsequently confirmed by Western blot. To diagnose chronic Chagas disease, a single serological test is not sufficiently sensitive and specific. Therefore, the standard procedure is to perform two or more tests that use different techniques such as ELISA and an immunofluorescence antibody test [13].

Additional histological examination of the post-LVAD myocardial biopsy showed no trypanosomes. *T. cruzi* PCR on the myocardial biopsy, also performed by the AMC, was negative, but positive for a blood specimen, albeit with a very low parasite load (crossing point value of 40).

## Diagnosis

Considering the patient’s clinical findings, the likelihood of being infected during his childhood in Surinam, the positive results of three distinct serological tests and a positive PCR result in blood for *T. cruzi*, a diagnosis of chronic Chagas disease with cardiac involvement was made. This corresponds with guidelines described by the Centers for Disease Control and Prevention concerning the diagnosis of Chagas disease [14]. Furthermore, no other cause of the cardiac complications and dilated cardiomyopathy was found.

## Treatment

Due to irreversible heart failure despite optimizing cardiac medication, cardiac transplantation was considered to be the only curative treatment option. The patient was placed on the waiting list for cardiac transplantation in January 2016 and the LVAD placed in September 2016 serves as a bridge to transplant. Trypanocidal therapy with benznidazole was not administered, as in this stage of the disease the beneficial effect on the clinical progression is limited and the drug can have considerable side effects such as dermatitis, gastro-intestinal complaints (e.g. vomiting and abdominal pain), polyneuritis and rarely bone marrow depression or toxic hepatitis [9, 15, 16].

## Outcome and follow-up

Currently, the patient is still on the waiting list for cardiac transplantation and it is not possible to predict when transplantation will take place. He returns to the outpatient clinic on a regular basis for check-up of his condition and his LVAD by the cardiologist. After transplantation he will be closely monitored for reactivation of Chagas disease which includes weekly PCR for *T. cruzi* on blood for the first 3 months, followed by PCR every 2 weeks in months 3–6 after transplantation and subsequently after every 6 months [17, 18]. The turnaround time of the PCR results is estimated to be 1 week because this test is performed in another laboratory (AMC). If clinical signs of reactivation are present, microscopic examination of blood smear preparations and quantitative buffy coat analysis will be performed immediately in our own laboratory to detect circulating parasites. Clinical signs of reactivation may include skin lesions, fever, feeling of malaise, swollen lymph nodes, hepatosplenomegaly, cardiac arrhythmias or neurological manifestations. Additional examinations depend on signs of clinical reactivation: if skin lesions are present, a skin biopsy can be done, endomyocardial biopsy can be considered if there is a suspicion of myocarditis and imaging of the brain and analysis of cerebrospinal fluid or brain biopsy may be performed when neurological complications occur [19–22]. When reactivation is confirmed by subsequent positive blood samples or positive biopsy (either PCR-positive for *T. cruzi* or confirmed presence of amastigotes in tissue), treatment with benznidazole will be started. Planned dosage is 5–7 mg kg^–1^ twice daily for at least 60 days [11, 16].

## Discussion

This report describes a patient with congestive heart failure due to Chagas disease, which is a rarely diagnosed infectious disease in the Netherlands. Nevertheless, infection with *T. cruzi* should be taken into account in patients with risk factors and corresponding clinical signs.

The pathophysiological mechanism underlying the chronic cardiac complications of Chagas disease is not entirely understood. A chronic inflammatory response elicited by *T. cruzi* leads to damage of the heart chambers and the conduction system. Several autoantigens cross react with *T. cruzi* antigens and antibodies, and are thought to play an important role in the immune-mediated tissue damage. However, parasite persistence is also believed to be essential for the development and progression of cardiac complications of chronic infection with *T. cruzi* [23, 24]. Intracellular amastigotes of *T. cruzi* are not sensitive to treatment with benznidazole. As a result, treatment is not likely to lead to eradication of all parasites during the chronic phase of the infection [23, 25, 26].

In a randomized trial by Morillo *et al*., benznidazole was compared to placebo in the treatment of patients with moderate to severe cardiomyopathy due to Chagas disease. Although parasite detection by PCR in blood was reduced, no significant reduction of clinical impairment was demonstrated [15].

Heart transplantation is the only treatment for patients with terminal cardiomyopathy due to chronic Chagas disease but it poses challenges due to the possibility of reactivation.

Reactivation of Chagas disease after heart transplantation is estimated to be between 19.6 and 45 %, although mortality of these patients due to reactivation is very low (0.9 %) [22, 27].

Taking the side effects of benznidazole and nifurtimox into consideration, use of these drugs in transplanted patients prior to or after transplantation is not unambiguous. In general, treatment of heart transplantation candidates with end-stage heart failure with benznidazole prior to transplantation is not supported by evidence in the literature [25].

The high-dose immunosuppressive agents prescribed after heart transplantation, particularly mycophenolate mofetil-based immunosuppression, results in an increased risk of *T. cruzi* reactivation. Therefore, it is advised that immunosuppression should be minimized and azathioprine should be used instead of mycophenolate mofetil [11, 25, 26, 28, 29]. In symptomatic patients, reactivation of *T. cruzi* is confirmed when trypomastigotes are detected in blood by microscopy or by quantitative buffy coat analysis, or when histological tissue examination shows inflammatory changes around tissue amastigotes. Studies have shown that prior to clinical signs of reactivation, PCR for *T. cruzi* on blood or tissue can become positive before circulating trypomastigotes can be identified by microscopy or histology. Almeida da Costa *et al*. retrospectively examined endomyocardial biopsy specimens from heart transplantation patients with Chagas disease. Patients who had the first clinical reactivation within 1 year after heart transplantation had the first positive endomyocardial biopsy within the first month if analysed by PCR for *T. cruzi* [19]. Diez *et al*. observed clinical reactivation on average 72 days after transplantation (range 38–92 days). However, two different PCR techniques on blood showed positive results on average 59 days before clinical manifestations (range 38–85 days) and 46 days before clinical reactivation (range 31–78 days). Most patients without clinical reactivation had a negative PCR for *T. cruzi* on blood samples [17]. This suggests that persistent parasitaemia is associated with increased risk of clinical reactivation. In chronic Chagas disease, parasitaemia can be low and intermittent. In the patient we described, PCR on blood was positive and represented a low load of circulating trypanosomes. Whether this indicates an increased risk for reactivation after heart transplantation has taken place is unknown. With early detection with PCR, anti-trypanosomal treatment can be started before clinical symptoms occur [17, 19, 22, 25]. However, PCR results should be interpretated with caution as these are influenced by the examined blood volumes. If small blood volumes are used, PCR detection levels decrease and results may be false-negative. Therefore, when clinical signs of reactivation are suspected, frequent screening by PCR in addition to microscopy or quantitative buffy coat analysis may enable early diagnosis of reactivation.

After transplantation the optimal timing of treatment with benznidazole is uncertain. Because we expect the PCR for *T. cruzi* on blood to become the first positive test in the case of reactivation, our intention is to start benznidazole after two subsequent positive blood samples, if allowed by the clinical condition of the patient.

The optimal management of heart transplantation candidates due to Chagas disease is not uniformly described due to the diversity of patients, lack of evidence and limited therapeutic options. Hence, further research on underlying pathophysiological mechanisms, new drugs and outcome of various clinical management strategies may lead to further optimization of treatment of advanced Chagas disease.
